# Distinct Serum MicroRNA Signatures and mRNA Decay Pathway Dysregulation in NSAID-Exacerbated Chronic Urticaria

**DOI:** 10.3390/ijms27020904

**Published:** 2026-01-16

**Authors:** Young-Min Ye, Jin Young Noh, Seung Ho Kim, Jiwon Yoon, Da-Hye Moon, Boyoun Choi, Se-Min Park, Kun-Woo Park, Jungmo Kim, Hyun Goo Woo

**Affiliations:** 1 Department of Allergy and Clinical Immunology, Ajou University School of Medicine, Suwon 16499, Republic of Korea; 2 Department of Biomedical Sciences, Graduate School of Ajou University, Suwon 16499, Republic of Korea; karlnoh0401@ajou.ac.kr (J.Y.N.);; 3 Department of Physiology, Ajou University School of Medicine, Suwon 16499, Republic of Korea; ksho7416@ajou.ac.kr; 4 Ajou Translational Omics Center (ATOC), Research Institute for Innovative Medicine, Ajou University Medical Center, Suwon 16499, Republic of Korea; 5 Clinical Trial Center, Ajou University Medical Center, Suwon 16499, Republic of Korea

**Keywords:** NSAID hypersensitivity, chronic spontaneous urticaria, microRNA, mRNA decay

## Abstract

Nonsteroidal anti-inflammatory drugs (NSAIDs) can exacerbate urticaria and/or angioedema in up to 30% of patients with chronic urticaria (CU), representing a distinct subtype characterized by heightened inflammation and leukotriene-driven pathophysiology. MicroRNAs (miRNAs) are post-transcriptional regulators that modulate immune and inflammatory responses. This study aimed to identify differentially expressed miRNAs (DEMs) according to NSAID hypersensitivity status and to elucidate their molecular networks in CU. Serum miRNA profiles were analyzed in 14 NSAID-exacerbated CU (NECU) and 16 NSAID-tolerant CU (NTCU) patients using an Affymetrix GeneChip^®^ miRNA 4.0 Array. DEMs were identified (fold difference > 1.5, *p* < 0.05), and validated targets were retrieved from the multiMiR database for network construction and Gene Ontology enrichment analyses. NECU patients exhibited a higher frequency of angioedema and systemic corticosteroid use than NTCU patients. Eight DEMs were identified, including upregulated miR-5001-5p, miR-4270, and miR-6869-5p, and downregulated miR-6511b-5p, miR-2277-5p, and miR-378h in NECU. Network integration revealed *AGO2-BTG2-LMNB2*, *NFIC-ZZZ3*, and *NUFIP2-GLG1* as central clusters, implicating dysregulation of mRNA decay and inflammatory signaling pathways. Reduced miR-6511b-5p expression may derepress *BRG1*, enhancing chromatin accessibility for inflammatory and leukotriene-synthetic genes. Distinct miRNA signatures differentiate NECU from NTCU, implying a miR-5001-5p/miR-6511b-5p–mRNA decay axis that links impaired post-transcriptional regulation with leukotriene-driven inflammation in CU. These findings highlight candidate miRNAs as potential biomarkers for disease endotyping and therapeutic stratification.

## 1. Introduction

Aspirin and other nonsteroidal anti-inflammatory drugs (NSAIDs) are widely used medications worldwide; however, they can induce hypersensitivity reactions in susceptible individuals [1]. In patients with chronic urticaria (CU), NSAIDs may trigger or exacerbate wheals and/or angioedema, a condition called NSAID-exacerbated chronic urticaria (NECU) according to the EAACI/ENDA (European Academy of Allergy and Clinical Immunology/European Network for Drug Allergy) guidelines [1,2]. Unlike NSAID-induced urticaria/angioedema, in which symptoms occur exclusively following NSAID exposure, NECU patients experience persistent urticarial symptoms with additional flares upon NSAID ingestion. Clinical studies have shown that up to 30% of CU patients exhibit NSAID hypersensitivity, making NECU one of the most prevalent subtypes of drug-exacerbated urticaria [3].

Compared to NSAID-tolerant CU (NTCU), patients with NECU tend to present with more severe disease activity, a higher frequency of angioedema, and a greater need for systemic corticosteroids or leukotriene receptor antagonists [4,5]. The underlying mechanisms of NECU are thought to involve cyclooxygenase-1 (COX-1) inhibition, resulting in an imbalance in arachidonic acid metabolism, reduced prostaglandin E2 (PGE2) production, and increased cysteinyl leukotriene synthesis [3,6,7]. However, these biochemical alterations alone do not fully explain the heterogeneous clinical manifestations or variable treatment responses observed among NECU patients.

Genetic studies have identified variants in genes involved in mast cell activation, histamine metabolism, and arachidonic acid pathways, including the high-affinity IgE receptor (*FCERIA*), histamine N-methyltransferase (*HNMT*), 5-lipoxygenase (*ALOX5*), cytosolic phospholipase A1 (*PLA2G4A*), leukotriene C4 synthase (*LTC4S*), and prostaglandin E2 receptor subtype EP4 (*PTGER4*) [8,9,10,11]. Consistently, higher atopy rates and elevated total IgE levels have been reported in NECU patients, implying a Th2-skewed immune milieu that may contribute to disease persistence and heightened susceptibility to NSAID-induced exacerbations [12].

In contrast to aspirin/NSAID-exacerbated respiratory disease, which is characterized by eosinophilic and autoimmune inflammation, NECU primarily reflects a mast-cell-driven, leukotriene-biased phenotype [3,13,14]. Nevertheless, the contribution of autoimmune mechanisms remains controversial, as reports on the prevalence of anti-thyroid antibodies or autologous serum skin test positivity in NECU have been inconsistent [15,16,17]. Therefore, NECU likely represents a heterogeneous endotype characterized by varying degrees of mast cell hyperreactivity and leukotriene-biased inflammation [15,18].

Recent research implies that post-transcriptional gene regulation by microRNAs (miRNAs) may contribute to the molecular heterogeneity of CU and modulate eicosanoid pathways [19,20,21]. miRNAs regulate gene expression through mRNA degradation or translational repression, thereby influencing cytokine signaling, cellular activation, and tissue inflammation [21]. Although dysregulated miRNA expression has been reported in CU [22,23,24,25], no study to date has comprehensively profiled serum miRNAs according to NSAID hypersensitivity status.

This study aimed to identify differentially expressed serum miRNAs (DEMs) between NECU and NTCU patients and to elucidate their regulatory networks and functional pathways, thereby providing novel insights into the molecular basis of NECU.

## 2. Results

### 2.1. Demographic and Clinical Characteristics

The demographic and clinical characteristics of patients with NECU and NTCU are presented in Table 1. There were no significant differences between the two groups in age, sex distribution, or disease severity at the time of sampling. However, the NECU group exhibited a significantly higher prevalence of angioedema (85.7% vs. 31.3%, *p* = 0.008), more frequent systemic corticosteroid use (92.9% vs. 43.8%, *p* = 0.007), and a greater need for leukotriene receptor antagonists (71.4% vs. 25.0%, *p* = 0.026), compared to the NTCU group. Patients with NECU also tended to receive up-dosed H1AHs more frequently than those with NTCU, although this difference was not statistically significant. The proportions of H1-antihistamine responders, omalizumab responders, and cyclosporine users were comparable between the two groups.

### 2.2. DEMs

Comparative analysis of serum miRNA expression profiles between patients with NECU and those with NTCU identified eight DEMs (Supplementary Table S1) that met the criteria of fold change > 1.5 and *p*-value < 0.05 (Table 2). Among these, five miRNAs were upregulated in NECU, including hsa-miR-3921, hsa-miR-6869-5p, hsa-miR-5001-5p, hsa-miR-4734, and hsa-miR-4270, while three miRNAs were downregulated, including hsa-miR-6511b-5p, hsa-miR-2277-5p, and hsa-miR-378b. The most prominent upregulation observed was for miR-4270 (2.17-fold, *p* = 0.029), whereas the greatest downregulation noted was for miR-6511b-5p (2.02-fold, *p* = 0.011).

**Figure 1:**
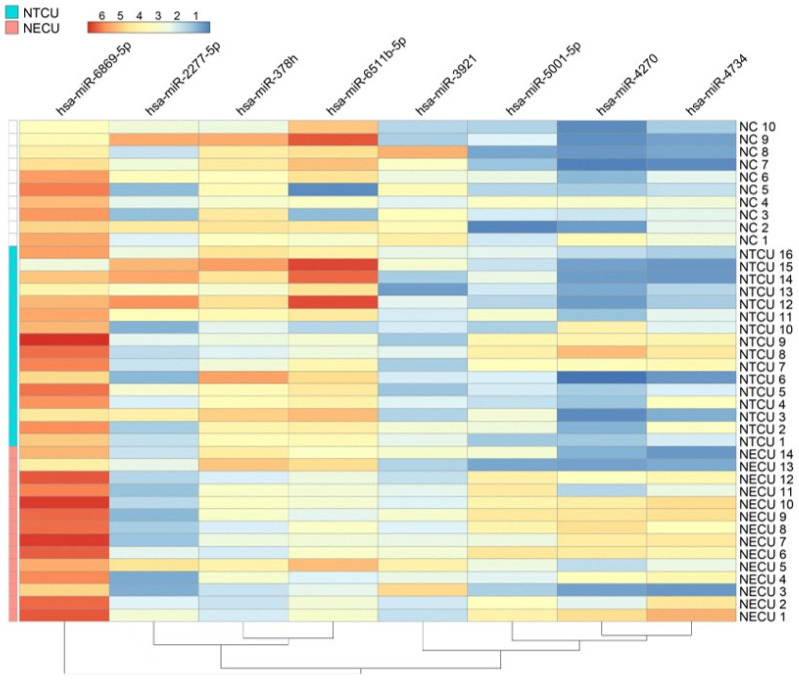
Heatmap of differentially expressed miRNAs between chronic urticaria patients according to aspirin/NSAID hypersensitivity. NECU, NSAID-exacerbated chronic urticaria; NTCU, NSAID-tolerant chronic urticaria; NC, healthy control.

Hierarchical clustering analysis based on these eight DEMs clearly distinguished NECU from NTCU samples (Figure 1). Distinct expression patterns were observed, with upregulated miRNAs (miR-6869-5p, miR-5001-5p, miR-4270, and miR-4734) showing higher expression in NECU, whereas downregulated miRNAs (miR-6511b-5p, miR-2277-5p, and miR-378b) exhibited lower expression levels in the same group.

### 2.3. Potential mRNA Targets of DEMs

Target mRNAs of the DEMs were identified from experimentally validated resources within the multiMiR package. Downstream analyses were limited to miRNAs with at least three validated target genes and to mRNAs supported by at least one database.

**Figure 2:**
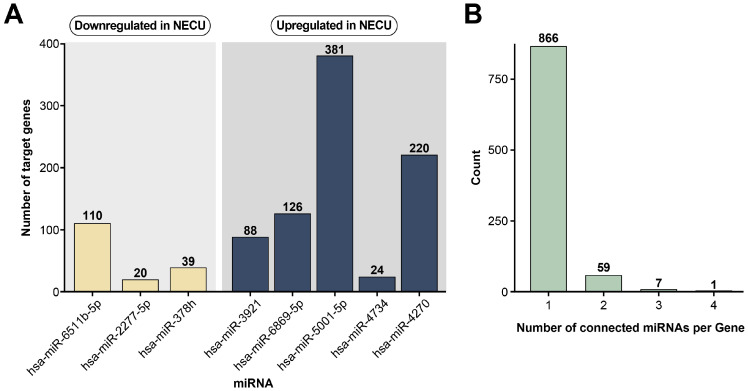
Identification of shared target genes of differentially expressed miRNAs distinguishing NECU from NTCU. (**A**) Numbers of experimentally validated target genes associated with the eight differentially expressed miRNAs were obtained using the multiMiR package, integrating results from TarBase v 9.0, miRTarBase v 9.0, and TargetScan v 8.0. (**B**) Putative target genes commonly regulated by three or more of these eight miRNAs were extracted, resulting in the identification of eight candidate hub genes (*AGO2*, *BTG2*, *LMNB2*, *NFIC*, *ZZZ3*, *NUFIP2*, *GLG1*, and *ZNF708*).

To elucidate the biological relevance of the DEMs distinguishing NECU from NTCU, experimentally validated target genes were identified using the multiMiR R package v2.4, which integrates data from miRTarBase v 9.0, TarBase v 9.0, and TargetScan v 9.0. Validated target genes were retrieved for each of the eight DEMs, and the number of targets varied substantially across miRNAs (Figure 2A). Among the upregulated miRNAs, miR-5001-5p exhibited the largest number of validated targets, whereas among the downregulated miRNAs, miR-6511b-5p and miR-2277-5p demonstrated fewer experimentally confirmed interactions.

To identify functionally convergent targets, putative genes regulated by three or more DEMs were extracted. This filtering yielded eight shared hub genes, including Argonaute 2 (*AGO2*), BTG anti-proliferation factor 2 (*BTG2*), Lamin B2 (*LMNB2*), Nuclear factor 1C (*NFIC*), ZZ-type zinc-finger-containing protein 3 (*ZZZ3*), Nuclear fragile X mental retardation protein-interacting protein 2 (*NUFIP2*), Golgi glycoprotein 1 (*GLG1*), and Zinc finger protein 707 (*ZNF708*), which were considered key regulatory nodes within the NECU-specific miRNA-mRNA network (Figure 2B).

**Figure 3:**
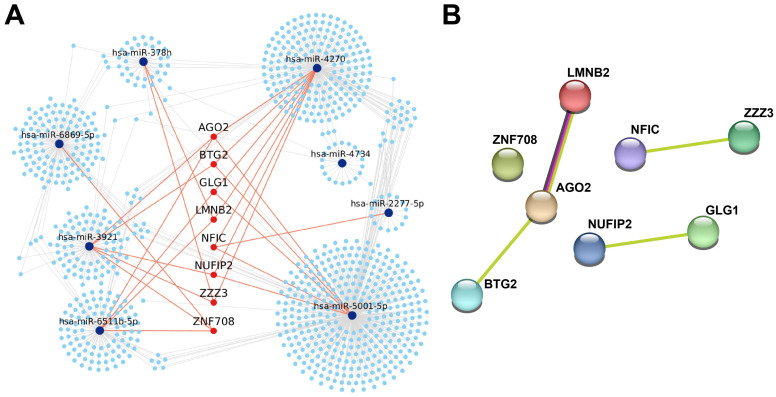
Interaction networks of validated target genes regulated by differentially expressed miRNAs in aspirin/NSAID-intolerant chronic urticaria. (**A**) Experimentally validated miRNA–mRNA interaction network constructed using TarBase v 9.0. Blue nodes represent differentially expressed miRNAs in aspirin/NSAID-intolerant chronic urticaria, and red nodes indicate eight hub genes. Red edges indicate interactions between miRNAs and hub genes, defined as target genes regulated by multiple differentially expressed miRNAs (≥3), whereas gray edges represent interactions with non-hub target genes. (**B**) PPI network of the identified hub genes generated using the STRING database (confidence score ≥ 0.4). *AGO2*, *BTG2*, and *LMNB2* formed the central interaction cluster, while *NFIC-ZZZ3* and *NUFIP2-GLG1* showed secondary connections, indicating potential cooperative functions in RNA processing, transcriptional regulation, and inflammatory signaling.

Network-based visualization using Cytoscape revealed that these hub genes were targeted by multiple DEMs, with *AGO2* representing the most central node in the interaction network (Figure 3A). The miR-5001-5p and miR-4270 subnetworks converged on *AGO2* and *BTG2*, core components of the miRNA decay machinery that is functionally associated with *LMNB2*, implying coordinated post-transcriptional regulation in NECU.

### 2.4. Functional Annotation of Hub Genes in NECU

To explore the biological functions of these hub genes, Gene Ontology (GO) Biological Process enrichment and protein–protein interaction (PPI) analyses were performed. The PPI network, generated using the STRING database (confidence score ≥ 0.15), revealed a central cluster composed of *AGO2*, *BTG2*, and *LMNB2*, with additional submodules including *NFIC-ZZZ3* and *NUFIP2-GLG1* (Figure 3B). These interactions imply potential cooperation in post-transcriptional gene silencing, chromatin remodeling, and translational regulation.

**Figure 4:**
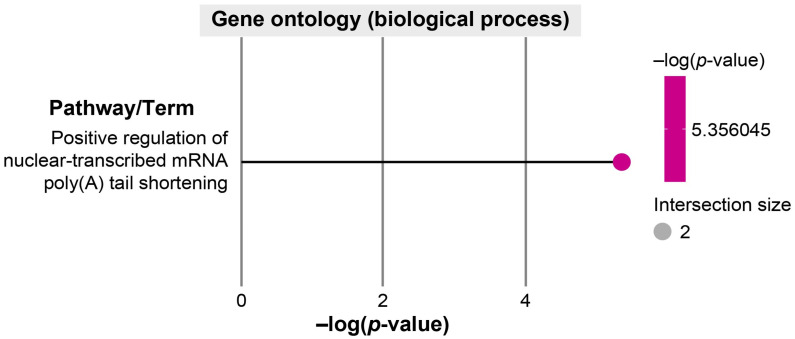
Gene Ontology (Biological Process) enrichment analysis of hub genes targeted by differentially expressed miRNAs in aspirin/NSAID-intolerant chronic urticaria. Functional enrichment analysis of eight putative hub genes commonly regulated by three or more miRNAs was performed using the Gene Ontology (Biological Process) category. Only one significantly enriched pathway, positive regulation of nuclear-transcribed mRNA poly(A) tail shortening, was identified, which participates in the *CCR4-NOT*-mediated mRNA deadenylation and decay complex. This finding suggests that impaired post-transcriptional mRNA degradation may contribute to sustained inflammatory gene expression in aspirin/NSAID-intolerant chronic urticaria.

GO enrichment analysis identified a single significantly enriched biological process—positive regulation of nuclear-transcribed mRNA poly(A) tail shortening (Figure 4). This pathway involves *AGO2*- and *BTG2*-mediated control of mRNA decay, implying that dysregulation of mRNA turnover may represent a key molecular feature in NECU.

Together, these results indicate that aberrant miRNA-mRNA interactions in NECU converge on pathways regulating mRNA stability, transcriptional control, and inflammatory mediator expression, providing mechanistic insights into the hyperreactive and leukotriene-biased inflammatory phenotype characteristics of NECU.

## 3. Discussion

This study identified distinct serum miRNA signatures that differentiate NECU from NTCU. Eight DEMs were detected, including upregulated expression of miR-3921, miR-6869-5p, miR-5001-5p, miR-4734, and miR-4270, and downregulated miR-6511b-5p, miR-2277-5p, and miR-378h. These miRNAs formed interconnected regulatory networks targeting *AGO2*, *BTG2*, and *LMNB2*. The hub genes were enriched in biological processes related to mRNA decay, transcriptional regulation, and inflammatory signaling, implying that post-transcriptional dysregulation may contribute to the heightened inflammatory reactivity observed in NECU. To our knowledge, this is the first study to profile circulating miRNAs according to aspirin/NSAID hypersensitivity status in CU, providing new insights into its molecular heterogeneity.

Although NSAID-exacerbated respiratory disease (NERD) has also been associated with distinct miRNA signatures in nasal mucosal samples, the key miRNAs reported in NERD differ from those identified in the present study [20]. This divergence likely reflects disease- and tissue-specific epigenetic regulation, underscoring that NECU represents a molecularly distinct NSAID-hypersensitivity phenotype rather than a cutaneous counterpart of NERD.

From a biological perspective, miRNAs function as fine-tuning regulators that modulate the magnitude, timing, and persistence of immune and inflammatory gene expression programs rather than acting as binary on–off switches for disease initiation [22,23,24]. Accordingly, the directionality of miRNA dysregulation observed in NECU likely reflects altered regulatory thresholds that shape inflammatory responsiveness following NSAID exposure. Downregulated miRNAs may permit sustained expression of genes involved in leukotriene synthesis, mast cell activation, and cellular stress adaptation, whereas upregulated miRNAs may represent compensatory responses to heightened inflammatory stress. Together, these findings suggest that miRNA dysregulation in NECU acts as a modulatory layer influencing disease severity and phenotype rather than initiating inflammation de novo.

CU patients exhibit increased infiltration of mast cells, basophils, eosinophils, and neutrophils in wheal lesions, all of which participate in arachidonic acid metabolism and cysteinyl leukotriene generation [26,27]. In NECU, preexisting urticarial inflammation is exacerbated following NSAID exposure, accompanied by elevated baseline and post-provocation urinary leukotriene E4 (LTE4) and increased serum tryptase levels, indicating enhanced mast-cell activation and leukotriene overproduction [13].

Pharmacologically, COX-1 inhibition reduces PGE2 synthesis, a key homeostatic mediator that normally limits mast-cell activation and vascular permeability. PGE2 acts through EP2 and EP4 receptors to increase intracellular cAMP and suppress IgE-mediated mast-cell degranulation [10]. Loss of this inhibitory tone after NSAID challenge results in excessive release of histamine, platelet-activating factor, and cytokines from mast cells. Concurrently, arachidonic acid metabolism is redirected toward the 5-lipoxygenase pathway, promoting overproduction of LTC4, LTD4, and LTE4. These changes amplify vascular permeability and inflammatory cell recruitment, driving the acute wheal and angioedema that occur more frequently in NECU than in NTCU patients.

Genetic predispositions may further enhance this exaggerated response. Variants in *ALOX5*, *LTC4S*, and *PLA2G4A* are associated with increased leukotriene synthesis, while promoter polymorphisms in *FCERIA* augment FcɛRIα expression and mast-cell sensitivity [8,9,11]. In addition, *PTGER4* promoter variants that reduce PGE2-EP4 signaling diminish the anti-inflammatory restraint normally imposed on mast cells [10]. Together, these mechanisms create a mast-cell-primed, leukotriene-dominant inflammatory milieu that underlies NECU pathogenesis.

Beyond its anti-inflammatory role, PGE2 also restrains platelet activation through EP4-mediated cAMP signaling [28]. Thus, decreased PGE2 availability after COX-1 blockade not only enhances mast-cell activation but also promotes platelet priming and platelet–leukocyte conjugate formation. This interaction gives rise to CD61+ platelet-adherent leukocytes, which correlate with urinary LTE4 levels during acute NSAID reactions [29]. Activated platelets release mediators that promote endothelial permeability and induce epithelial alarmins, such as IL-33 and thymic stromal lymphopoietin [30,31]. These alarmins, in turn, sensitize mast cells and basophils, establishing a feed-forward inflammatory loop that promotes vascular leakage. Unlike NSAID-exacerbated respiratory disease, in which systemic platelet activation and eosinophil-platelet complexes persist at baseline, NECU maintains normal platelet homeostasis, with activation occurring transiently after COX-1 inhibition [29].

Our results further imply that these immunometabolic pathways are modulated by miRNA-mediated post-transcriptional regulation. Inflammation-related miRNAs, such as miR-146a, miR-155, and miR-21, have been reported to regulate enzymes in the eicosanoid biosynthetic cascade, including 5-lipoxygenase-activating protein and COX-2 (*PTGS2*), thereby influencing leukotriene synthesis [21]. Clinically, this mechanistic link is supported by the favorable efficacy of leukotriene receptor antagonists in patients with NECU [32].

Although members of the miR-378 family have been reported to regulate *PTGS2 (COX-2)* expression, its reduced expression in NECU may release this inhibition, leading to COX-2 upregulation [21]. However, impaired PGE2-EP4 signaling and an altered prostanoid–leukotriene balance likely prevent COX-2-derived PGE2 from exerting its anti-inflammatory effects, thereby favoring leukotriene-dominant inflammation.

Several miRNAs previously reported to be upregulated in CSU, including miR-2355-3p, miR-4264, miR-2355-5p, miR-29c-5p, and miR-361-3p, were not identified as differentially expressed in the present analysis [24,25]. These miRNAs have mainly been implicated in general inflammatory processes in CU, such as cytokine signaling and leukocyte activation, based on comparisons between CSU patients with healthy controls [22,23,24,25]. In contrast, our study focused on NSAID hypersensitivity-associated miRNA alterations within a homogeneous CU population, supporting NECU as a distinct molecular endotype rather than an extension of baseline CSU inflammation.

Network-based analyses identified *AGO2*, *BTG2*, and *LMNB2* as hub genes, implying dysfunction of the mRNA poly(A)-tail shortening and decay machinery. *AGO2* functions as the catalytic core of the RNA-induced silencing complex [33], while *BTG2* cooperates with the *CCR4-NOT* complex to promote mRNA deadenylation [34,35]. Disruption of this regulatory axis may prolong the lifespan of inflammatory transcripts, linking altered miRNA expression with sustained cytokine and lipid mediator production in NECU.

Another important finding was the potential involvement of the miR-6511b-5p-BRG1-CD44 regulatory axis and its functional link to the osteopontin-CD44 pathway [36,37]. miR-6511b-5p has been reported to repress CD44 expression indirectly by targeting *BRG1*, leading to epigenetic silencing of the *CD44* gene [36]. As osteopontin (*SPP1*) binds to its receptor CD44 to promote mast-cell adhesion, chemotaxis, and degranulation [37], reduced miR-6511b-5p expression may enhance both CD44 availability and osteopontin-mediated inflammatory signaling. Elevated plasma osteopontin and increased dermal expression have been reported in patients with CU, supporting its role in mast-cell-mediated inflammation [37,38]. Thus, miR-6511b-5p downregulation in NECU may reinforce the osteopontin–CD44 axis, augmenting mast-cell activation and leukotriene-mediated inflammation.

Together, our findings extend the pathogenic model of NECU beyond COX-1 inhibition and genetic predisposition, implying a multilayered regulatory framework that integrates PGE2 deficiency, platelet–leukocyte interaction, and miRNA-mediated post-transcriptional dysregulation. The integration of the miR-378h-PTGS2, *AGO2-BTG2-LMNB2*, and miR-6511b-5p-CD44-osteopontin networks provides a conceptual link between mRNA stability, eicosanoid metabolism, and mast-cell activation in NECU. These insights highlight circulating miRNAs as potential biomarkers reflecting disease endotypes and therapeutic responsiveness.

This study has several limitations. First, the sample size was relatively small, and validation in a larger, independent cohort is warranted. Second, functional assays were not performed; therefore, the proposed miRNA–mRNA interactions remain hypothetical. Finally, although circulating miRNAs do not provide direct cell- or tissue-specific spatial resolution, they represent stable, biologically active regulators that integrate systemic immune and inflammatory signals. Given the systemic nature of leukotriene-driven inflammation in NECU, serum miRNA profiling offers a clinically relevant and translationally accessible approach. Future studies integrating serum miRNA signatures with tissue- and cell-specific transcriptomic or proteomic analyses may further refine the mechanistic interpretation of these regulatory networks.

## 4. Materials and Methods

### 4.1. Study Participants and Clinical Assessment

In total, 30 patients with chronic spontaneous urticaria were recruited from the Department of Allergy and Clinical Immunology at a university hospital in Korea between January and May 2018. Patients were categorized into two groups according to their clinical response to NSAIDs. The NECU group (*n* = 14) included patients with a documented history of recurrent urticaria and/or angioedema exacerbated by NSAID or aspirin intake, or those who showed a positive response to an NSAID oral provocation test. The NTCU group (*n* = 16) comprised patients with no history of NSAID-induced symptom aggravation and confirmed negative results on oral provocation testing.

All subjects met the diagnostic criteria for chronic spontaneous urticaria, defined as recurrent wheals and/or angioedema persisting for more than 6 weeks. Disease severity was evaluated using the Urticaria Activity Score over 7 days, the Urticaria Control Test, and the presence of angioedema. Information on medication use, including H1-antihistamines, systemic corticosteroids, cyclosporine, and omalizumab, was obtained from medical records.

The study protocol was reviewed and approved by the Institutional Review Board of Ajou University Medical Center (AJOUIRB-SMP-2019-332 and approval date: 18 October 2019), and written informed consent was obtained from all participants prior to enrollment.

### 4.2. Serum Preparation and miRNA Extraction

Peripheral blood samples were collected from all participants at the time of enrollment before any modification in treatment. Serum was isolated by centrifugation at 3000× *g* for 10 min and stored at −80 °C until analysis. Total miRNAs were extracted from 100 μL of serum using an miRNeasy Serum/Plasma Kit (Qiagen, Hilden, Germany) according to the manufacturer’s instructions. Synthetic Caenorhabditis elegans miR-39-3p (Cel-39-3p) was spiked into each sample as an internal control for normalization.

### 4.3. miRNA Microarray and Data Analysis

Serum miRNA expression profiling was performed using an Affymetrix GeneChip^®^ miRNA 4.0 Array (Homo sapiens), which contains 2578 probes for mature human miRNAs. Microarray hybridization and scanning were conducted at Macrogen, Inc. (Seoul, Republic of Korea) following the manufacturer’s protocol. Raw microarray data were processed using Transcriptome Analysis Console software v4.0.2 (Thermo Fisher Scientific, Waltham, MA, USA), including background correction and normalization.

DEMs between the NECU and NTCU groups were identified using a fold difference > 1.5 and *p* < 0.05 (unpaired two-tailed *t*-test). For visualization, normalized miRNA expression values were transformed to row-wise z-scores, and hierarchical clustering was performed using Euclidean distance with complete linkage. Given the exploratory nature of this study and the limited sample size, false discovery rate correction was not applied, and *p* values are presented descriptively.

### 4.4. Target Gene Prediction and Functional Analysis

Validated target genes for each DEM were identified using the multiMiR database (version 2.4) in Bioconductor (version 1.22.2). Only target genes with experimentally validated interactions supported by at least one curated database (e.g., miRTarBase, TarBase, or miRecords) were retained for downstream analysis.

miRNA-mRNA interaction networks were visualized using Cytoscape (version 3.10.1). GO enrichment analysis was performed using g:Profiler2 (version 0.2.4; (https://biit.cs.ut.ee/gprofiler/page/r, accessed on 1 August 2025). PPI networks were generated using STRING (version 12.0; https://string-db.org, accessed on 25 September 2025), applying a combined confidence score ≥ 0.15.

### 4.5. Statistical Analysis

Clinical and laboratory variables are presented as means ± standard deviation (SD) or as numbers (percentages), as appropriate. Given the limited sample size, normality of clinical variables was not assumed. Accordingly, continuous variables were compared between the NECU and NTCU groups using the Mann–Whitney U test, and categorical variables were analyzed using Fisher’s exact test. Differential miRNA expression between the two groups was assessed using unpaired two-tailed *t*-tests applied to normalized microarray expression data, consistent with standard exploratory microarray analyses. All statistical analyses were performed using IBM SPSS Statistics for Windows, version 29.0 (IBM Corp., Armonk, NY, USA). A two-tailed *p*-value of <0.05 was considered statistically significant.

## 5. Conclusions

NECU is characterized by constitutive leukotriene overproduction from primed mast cells and transient platelet activation following the loss of PGE2-mediated inhibition after COX-1 blockade. Alterations in miR-378h, miR-5001-5p, and miR-6511b-5p, together with dysregulation of the *AGO2-BTG2-LMNB2* axis, are likely to contribute to persistent inflammatory activation and impaired post-transcriptional regulation. These findings provide novel mechanistic insights into the pathogenesis of NECU and highlight circulating miRNAs as potential biomarkers and therapeutic targets for precision management of the disease.

## Figures and Tables

**Table 1:** Comparison of clinical features between NECU and NTCU.

Features	NECU (*n* = 14)	NTCU (*n* = 16)	*p*-Value
Female sex (%)	9 (64.3)	6 (37.5)	0.272
Age (years)	37.1 ± 9.8	38.6 ± 10.1	0.700
Presence of angioedema (%)	12 (85.7)	5 (31.3)	0.008
UAS7	31.8 ± 8.7	28.1 ± 12.3	0.501
H1AH responders (%)	5 (35.7)	8 (50.0)	0.676
Up-dosed H1AH (%)	10 (71.4)	6 (37.5)	0.081
LTRA use (%)	10 (71.4)	4 (25.0)	0.026
Omalizumab responders (%)	4/8 (50.0)	5/7 (71.4)	0.608
Systemic steroid use (%)	13 (92.9)	7/6 (43.8)	0.007
Cyclosporine use (%)	5 (35.7)	4 (25.0)	0.694

NECU, NSAID-intolerant chronic urticaria; NTCU, NSAID-tolerant chronic urticaria; UAS7, urticaria activity score over 7 days; H1AH, H1-antihistamine; Up-dosed, loratadine-equivalent > 20 mg/day; LTRA, leukotriene receptor antagonist.

**Table 2:** Differential miRNA expression between aspirin-intolerant and aspirin-tolerant chronic urticaria patients.

miRNA	NECU (*n* = 14)	NTCU (*n* = 16)	Fold Difference	*p*-Value
hsa-miR-3921	2.80 ± 0.8	2.19 ± 0.6	1.53 increase	0.025
hsa-miR-6869-5p	5.73 ± 0.7	5.09 ± 0.9	1.56 increase	0.047
hsa-miR-5001-5p	3.26 ± 1.0	2.61 ± 0.7	1.57 increase	0.040
hsa-miR-4734	3.27 ± 1.5	2.32 ± 1.2	1.94 increase	0.029
hsa-miR-4270	3.00 ± 1.4	1.88 ± 1.4	2.17 increase	0.029
hsa-miR-6511b-5p	3.25 ± 0.7	4.26 ± 1.2	2.02 decrease	0.011
hsa-miR-2277-5p	2.07 ± 0.9	2.98 ± 1.4	1.87 decrease	0.048
hsa-miR-378h	3.01 ± 0.9	3.81 ± 0.9	1.74 decrease	0.018

NECU, NSAID-intolerant chronic urticaria; NTCU, NSAID-tolerant chronic urticaria. Differential expression was defined as a fold difference of >1.5 and *p*-value of <0.05 when comparing miRNA expression.

## Data Availability

The original contributions presented in this study are included in the article/Supplementary Materials. Further inquiries can be directed to the corresponding author.

## Supplementary Materials

The following supporting information can be downloaded at: https://www.mdpi.com/article/10.3390/ijms27020904/s1.

## Abbreviations

CU	Chronic urticaria
NSAIDs	Nonsteroidal anti-inflammatory drugs
NECU	NSAID-exacerbated chronic urticaria
NTCU	NSAID-tolerant CU
miRNA	MicroRNA
DEMs	Differentially expressed miRNAs
GO	Gene Ontology
AGO2	Argonaute 2
BTG2	BTG anti-proliferation factor 2
LMNB2	Lamin B2
COX-1	Cyclooxygenase-1
PGE2	Prostaglandin E2
NFIC	Nuclear factor 1C
ZZZ3	ZZ-type zinc-finger-containing protein 3
NUFIP2	Nuclear fragile X mental retardation protein-interacting protein 2
GLG1	Golgi glycoprotein 1

## References

[1] Kowalski M.L., Asero R., Bavbek S., Blanca M., Blanca-Lopez N., Bochenek G., Brockow K., Campo P., Celik G., Cernadas J. (2013). Classification and practical approach to the diagnosis and management of hypersensitivity to nonsteroidal anti-inflammatory drugs. Allergy.

[2] Kowalski M.L., Makowska J.S. (2015). Seven steps to the diagnosis of NSAIDs hypersensitivity: How to apply a new classification in real practice?. Allergy Asthma Immunol. Res..

[3] Laidlaw T.M., Cahill K.N. (2017). Current knowledge and management of hypersensitivity to aspirin and NSAIDs. J. Allergy Clin. Immunol. Pract..

[4] Rebelo Gomes E., Geraldes L., Gaspar Â., Malheiro D., Cadinha S., Abreu C., Chambel M., Almeida E., Faria E., Portuguese Society of Allergology and Clinical Immunology (SPAIC) Drug Allergy Interest Group (2016). Hypersensitivity reactions to nonsteroidal anti-inflammatory drugs among adults: Clinical features and risk factors for diagnosis confirmations. Int. Arch. Allergy Immunol..

[5] Ye Y.M., Kim J.E., Nahm D.I., Kim S.H., Suh C.H., Nahm D.H., Park H.S. (2005). Comparison of clinical characteristics and prognosis of chronic urticaria according to the aspirin sensitivity. Korean J. Asthma Allergy Clin. Immunol..

[6] Mastalerz L., Setkowicz M., Szczeklik A. (2005). Mechanism of chronic urticaria exacerbation by aspirin. Curr. Allergy Asthma Rep..

[7] Asero R. (2022). Nonsteroidal anti-inflammatory drugs hypersensitivity in chronic spontaneous urticaria in the light of its pathogenesis. Eur. Ann. Allergy Clin. Immunol..

[8] Losol P., Yoo H.S., Park H.S. (2014). Molecular genetic mechanisms of chronic urticaria. Allergy Asthma Immunol. Res..

[9] Palikhe N.S., Kim S.H., Park H.S. (2008). What do we know about the genetics of aspirin intolerance?. J. Clin. Pharm. Ther..

[10] Palikhe N.S., Sin H.J., Kim S.H., Sin H.J., Hwang E.K., Ye Y.M., Park H.S. (2012). Genetic variability of prostaglandin E2 receptor subtype EP4 gene in aspirin-intolerant chronic urticaria. J. Hum. Genet..

[11] Bae J.S., Kim S.H., Ye Y.M., Yoon H.J., Suh C.H., Nahm D.H., Park H.S. (2007). Significant association of FcepsilonRIalpha promoter polymorphisms with aspirin-intolerant chronic urticaria. J. Allergy Clin. Immunol..

[12] Ye Y.M., Hur G.Y., Park H.J., Kim S.H., Kim H.M., Park H.S. (2008). Association of specific IgE to staphylococcal superantigens with the phenotype of chronic urticaria. J. Korean Med. Sci..

[13] Zembowicz A., Mastalerz L., Setkowicz M., Radziszewski W., Szczeklik A. (2003). Safety of cyclooxygenase 2 inhibitors and increased leukotriene synthesis in chronic idiopathic urticaria with sensitivity to nonsteroidal anti-inflammatory drugs. Arch. Dermatol..

[14] Woo S.D., Luu Q.Q., Park H.S. (2020). NSAID-exacerbated respiratory disease (NERD): From pathogenesis to improved care. Front. Pharmacol..

[15] Ye Y.M., Park J.W., Kim S.H., Ban G.Y., Kim J.H., Shin Y.S., Lee H.Y., Park H.S., PRANA Group (2016). Prognostic factors for chronic spontaneous urticaria: A 6-month prospective observational study. Allergy Asthma Immunol. Res..

[16] Asero R., Tedeschi A., Lorini M. (2002). Autoreactivity is highly prevalent in patients with multiple intolerances to NSAIDs. Ann. Allergy Asthma Immunol..

[17] Erbagci Z. (2004). Multiple NSAID intolerance in chronic idiopathic urticaria is correlated with delayed, pronounced and prolonged autoreactivity. J. Dermatol..

[18] Shin Y.S., Suh D.H., Yang E.M., Ye Y.M., Park H.S. (2015). Serum specific IgE to thyroid peroxidase activates basophils in aspirin intolerant urticaria. J. Korean Med. Sci..

[19] Karstarli Bakay O.S., Demir B., Cicek D., Erol D., Toraman Z.A., Gural Y., Maurer M. (2023). In chronic spontaneous urticaria, IgE and C-reactive protein are linked to distinct microRNAs and interleukin-31. Clin. Transl. Allergy.

[20] Gajewski A., Bekier A., Frachowicz-Guereirro K., Drożdż I., Ćwikliński R., Kurowski M., Kowalski M.L., Baumann R., Schmidt-Weber C., Chaker A.M. (2025). Analysis of miRNA expression in patients with NSAID-exacerbated respiratory disease. Allergy Asthma Immunol. Res..

[21] Saul M.J., Emmerich A.C., Steinhilber D., Suess B. (2019). Regulation of eicosanoid pathways by microRNAs. Front. Pharmacol..

[22] Deng Q., Yao X., Fang S., Sun Y., Liu L., Li C., Li G., Guo Y., Liu J. (2025). Mast cell-mediated microRNA functioning in immune regulation and disease pathophysiology. Clin. Exp. Med..

[23] Dopytalska K., Czaplicka A., Szymańska E., Walecka I. (2023). The essential role of microRNAs in inflammatory and autoimmune skin diseases—A review. Int. J. Mol. Sci..

[24] Brancaccio R., Murdaca G., Casella R., Loverre T., Bonzano L., Nettis E., Gangemi S. (2023). miRNAs’ cross-involvement in skin allergies: A new horizon for the pathogenesis, diagnosis and therapy of atopic dermatitis, allergic contact dermatitis and chronic spontaneous urticaria. Biomedicines.

[25] Lin C.K.E., Kaptein J.S., Sheikh J. (2017). Differential expression of microRNAs and their possible roles in patients with chronic idiopathic urticaria and active hives. Allergy Rhinol..

[26] Giménez-Arnau A., Curto-Barredo L., Nonell L., Puigdecanet E., Yelamos J., Gimeno R., Rüberg S., Santamaria-Babi L., Pujol R.M. (2017). Transcriptome analysis of severely active chronic spontaneous urticaria shows an overall immunological skin involvement. Allergy.

[27] Ying S., Kikuchi Y., Meng Q., Kay A.B., Kaplan A.P. (2002). TH1/TH2 cytokines and inflammatory cells in skin biopsy specimens from patients with chronic idiopathic urticaria: Comparison with the allergen-induced late-phase cutaneous reaction. J. Allergy Clin. Immunol..

[28] Friedman E.A., Ogletree M.L., Haddad E.V., Boutaud O. (2015). Understanding the role of prostaglandin E2 in regulating human platelet activity in health and disease. Thromb. Res..

[29] Jurado-Escobar R., Doña I., Bogas-Herrera G., Pérez-Sánchez N., Salas M., Laguna J.J., Muñoz-Cano R., Mayorga C., Torres M.J., Cornejo-García J.A. (2020). Platelet-adherent leukocytes associated with cutaneous cross-reactive hypersensitivity to nonsteroidal anti-inflammatory drugs. Front. Pharmacol..

[30] Dobrican-Băruța C.T., Deleanu D.M., Muntean I.A., Nedelea I., Bălan R.G., Filip G.A., Procopciuc L.M. (2024). The alarmin triad—IL-25, IL-33, and TSLP—Serum levels and their clinical implications in chronic spontaneous urticaria. Int. J. Mol. Sci..

[31] Choi B.Y., Ye Y.M. (2024). Role of platelet-activating factor in the pathogenesis of chronic Spontaneous Urticaria. Int. J. Mol. Sci..

[32] Rayner D.G., Liu M., Chu A.W.L., Chu X., Guyatt G.H., Oykhman P., Cao D.J., Moellman J., Ben-Shoshan M., Baker D.R. (2024). Leukotriene receptor antagonists as add-on therapy to antihistamines for urticaria: Systematic review and meta-analysis of randomized clinical trials. J. Allergy Clin. Immunol..

[33] Meister G., Tuschl T. (2004). Mechanisms of gene silencing by double-stranded RNA. Nature.

[34] Pavanello L., Hall M., Winkler G.S. (2023). Regulation of eukaryotic mRNA deadenylation and degradation by the Ccr4-Not complex. Front. Cell Dev. Biol..

[35] Doidge R., Mittal S., Aslam A., Winkler G.S. (2012). Deadenylation of cytoplasmic mRNA by the mammalian Ccr4-Not complex. Biochem. Soc. Trans..

[36] Sun J., Ye L., Shi Y., Wang X., Zhao X., Ren S., Fan J., Shao H., Qin B. (2022). MiR-6511b-5p suppresses metastasis of pMMR colorectal cancer through methylation of CD44 by directly targeting BRG1. Clin. Transl. Oncol..

[37] Topsakal M., Kaçar N., Demirkan N., Aybek H., Tosun Yıldırım H., İmren I.G., Özden M.G. (2020). Osteopontin in chronic urticaria: Elevated plasma levels and significantly increased osteopontin expression in patients’ skin samples compared to controls. Dermatol. Ther..

[38] Nagasaka A., Matsue H., Matsushima H., Aoki R., Nakamura Y., Kambe N., Kon S., Uede T., Shimada S. (2008). Osteopontin is produced by mast cells and affects IgE-mediated degranulation and migration of mast cells. Eur. J. Immunol..

