# Supplementary material for: Distinct Serum MicroRNA Signatures and mRNA Decay Pathway Dysregulation in NSAID-Exacerbated Chronic Urticaria
Source: Int J Mol Sci. 2026 Jan 16;27(2):904. doi: 10.3390/ijms27020904 (PMC12842516; doi:10.3390/ijms27020904)
Supplement: Supplementary file 1 [file ijms-27-00904-s001.zip › ijms-4057875-supplementary.pdf]

Supplementary Table S1. Sequence of selected miRNA and the number of proposed target genes in various miRNA databases.

miRNA	miRNA sequence	Reported target genes (N)			
		Tb	microT	miRDB	TS
hsa-miR-3921	UCUCUGAGUACCAUAUGCCUUGU	3	84	2,376	312
hsa-miR-6869-5p	GUGAGUAGUGGCGCGCGGCGGC	2	71	1,953	248
hsa-miR-5001-5p	AGGGCUGGACUCAGCGGCGGAGCU	5	96	2,104	275
hsa-miR-4734	GCUGCGGGCUGCGGUCAGGGCG	1	69	1,785	241
hsa-miR-4270	UCAGGGAGUCAGGGGAGGGC	4	80	1,932	257
hsa-miR-6511b-5p	CUGCAGGCAGAAGUGGGGCUGACA	2	73	1,698	221
hsa-miR-2277-5p	AGCGCGGGCUGAGCGCUGCCAGUC	6	88	2,021	269
hsa-miR-378h	ACUGGACUUGGUGUCAGAUGG	9	102	2,477	286

Tb, Tarbase v 9.0; microT, DIANA-microT-CDS v 5.0; miR DB, miRDB v 6.0; TS, TargetScan v 8.0
